# Comprehensive insights into the effects and regulatory mechanisms of immune cells expressing programmed death-1/programmed death ligand 1 in solid tumors

**DOI:** 10.20892/j.issn.2095-3941.2020.0112

**Published:** 2020-08-15

**Authors:** Min Liu, Qian Sun, Feng Wei, Xiubao Ren

**Affiliations:** ^1^Department of Immunology, Tianjin Medical University Cancer Institute and Hospital, National Clinical Research Center for Cancer, Key Laboratory of Cancer Prevention and Therapy, Tianjin, Key Laboratory of Cancer Immunology and Biotherapy, Tianjin’s Clinical Research Center for Cancer, Tianjin 300060, China

**Keywords:** Immune cell, immunotherapy, programmed cell death ligand 1, programmed cell death-1, solid tumor

## Abstract

The programmed cell death-1 (PD-1)/programmed cell death ligand 1 (PD-L1) signaling pathway is an important mechanism in tumor immune escape, and expression of PD-L1 on tumor cells has been reported more frequently. However, accumulating evidence suggests that PD-1/PD-L1 is also widely expressed on immune cells, and that regulation is also critical for tumor immune responses. In this review, we emphasized that under solid tumor conditions, the immunoregulatory effects of immune cells expressing PD-1 or PD-L1, affected the prognoses of cancer patients. Therefore, a better understanding of the mechanisms that regulate PD-1 or PD-L1 expression on immune cells would provide clear insights into the increased efficacy of anti-PD antibodies and the development of novel tumor immunotherapy strategies.

## Introduction

Programmed cell death ligand 1 (PD-L1) expression on tumor cells is often observed in human cancers, and its binding to the programmed cell death-1 (PD-1) on activated T cells inhibits T cell proliferation, survival, cytokine production, and other effector functions, and ultimately inhibits anti-tumor immune responses^1,2^. Anti-PD antibodies show encouraging durable response rates, survival, and tolerability, supporting its therapeutic use in many types of cancers^3^. Use of anti-PD antibodies reverses functional inhibition of some immune cells and restores potent antitumor cytotoxic activity^4,5^. Therefore, the expression of PD-L1/PD-1 in the tumor microenvironment has important clinical significance.

In addition to tumor cells, the expression of PD-L1/PD-1 on immune cells is also very widespread, and contributes to a complicated network between immune cells and tumor cells, which is crucial for the curative effects and resistance mechanisms of anti-PD antibodies^6^. However, the role of PD-1^+^ or PD-L1^+^ immune cells in tumor immunity and the mechanisms by which they exert their effects remain largely unknown. In this review, we therefore focused on the biological function of immune cells expressing PD-1 or PD-L1, and summarized their interactions with other immune or tumor cells. The mechanisms that regulate PD-1 or PD-L1 expression in immune cells were also discussed. In addition, we described the potential therapeutic strategies of anti-PD-L1/PD-1 therapies, which provide new ideas and directions for improving the efficacy of anti-PD antibodies and developing new combined immunotherapy strategies.

## Immune cells with high expression of PD-1 or PD-L1 help predict tumor progression and prognosis

### T cells

The expression of PD-1 is absent or cannot be detected on naive T cells^7^. During cancer, PD-1 is upregulated on activated T cells to induce immune tolerance^8^. Many studies have indicated that increased PD-1^+^CD8^+^ T cell levels are associated with poorer prognosis in solid tumors^9–12^, including liver, pancreatic, head and neck cancer (HNC), non-small cell lung cancer, and early breast cancer. However, a recent study^13^ reported that PD-1^+^CD8^+^ immune subsets are associated with improved survival in triple-negative breast cancer (TNBC), while the CD8^-^PD-1^+^ immune subset was not associated. They found that some T cells express PD-1 but do not express other markers associated with exhaustion, and these cells produce proinflammatory cytokine levels similar to those of effector T cells. Therefore, PD-1 expression status alone does not distinguish between exhausted and activated T cells. This association is determined by the integrated results of epigenetic T cell receptor (TCR) signal intensity and epigenetics of the tumor microenvironment.

### B cells

Xiao et al.^14^ identified a new group of tumorigenic PD-1^hi^ B cells in liver cancer. High infiltration of PD-1^hi^ B cells is associated with disease stage and early recurrence in patients. Triggering PD-1 signaling induces PD-1^hi^ B cells with the ability to inhibit tumor-specific immunity and promote disease progression. For PD-L1 expression, B cells from the healthy control group showed little expression of PD-L1, B-cells from patients with stage I and II melanoma showed detectable low PD-L1, and naive B cells from stage III and IV showed moderate PD-L1. Moreover, compared with primary tumors, PD-L1^+^ B cells were upregulated in advanced melanomas and enriched in metastases^15^. Overall, high expression of PD-L1 on B cells is generally considered to be one of the characteristics of immunoregulatory functions that inhibit T cell proliferation and promote tumor progression in many types of tumors^16^.

### Natural killer cells (NK cells)

PD-1^+^ NK cells were also found in patients with digestive cancers, including colorectal cancer, gastric cancer (GC), esophageal squamous-cell carcinoma (ESCC), hepatocellular carcinoma (HCC), and biliary cancer^17^. In ESCC and HCC, increased PD-1 expression on NK cells is related to a poor prognosis^17^. However, more PD-1^+^ NK cells in the circulation are associated with better prognosis in patients with HNC^18^. Consistently, compared to PD-1^−^ NK cells, PD-1^+^ NK cells exhibit an activated state and highly expressed CD107a and NKG2D, which increase the expression of interferon (IFN)-γ and CD107a by PD-1 blockade^18^.

### Dendritic cells (DCs)

In ovarian cancer, in addition to B7-H1, tumor-associated DCs gradually express elevated levels of PD-1 over time. These double-positive PD-1^+^PD-L1^+^ DCs have a classical DC phenotype, but they are immature, highly inhibitory and have a poor response to risk^19^. The PD-1^+^PD-L1^+^ DCs use this well-known PD-L1/PD-1 inhibitory pathway to mediate immunosuppression and promote tumor progression.

### Mononuclear macrophages

Gordon et al.^20^ found that both mouse and human tumor-associated macrophages (TAMs) express PD-1 in colorectal cancer. PD-1 expression by TAMs increases over time and with the stage of disease. Earlier studies in liver cancer found that PD-L1^+^ monocytes accumulate in the stromal region surrounding the tumor and increase with tumor progression in HCC^21^. Subsequently, Herbst et al.^22^ found that macrophages expressing PD-L1 are more abundant in several tumors than tumor cells expressing PD-L1, and their density is related to the efficacy of anti-PD antibodies. There is increasing evidence that TAM-expressing PD-L1 contributes to immunosuppressive tumor microenvironments and is associated with poor clinical outcomes^23^. As a result, further characterization of the role of PD-1 and PD-L1 expression in regulating the biological functions of macrophages, especially TAM, is of great significance for understanding the blocking effect of immune checkpoint inhibitors and selecting the best combination of immunotherapies.

### Granulocytes

There is significantly increased neutrophil infiltration in tumor tissues of patients with GC, and these tumor-infiltrating neutrophils have an activated CD54^+^ phenotype and express high levels of PD-L1, and are related to the disease stage and the survival rate of patients with GC^24^. A recent study found that mast cell levels increased with tumor progression and could independently predict a decrease in overall survival of GC patients^25^. More importantly, these tumor-induced mast cells strongly expressed PD-L1 in a time- and dose-dependent manner, suggesting that these mast cells may be novel targets in novel GC treatments.

### Myeloid-derived suppressor cells (MDSCs)

In the 4T1 breast cancer mouse model, both spleen- and tumor-derived MDSCs express PD-1. The expression of PD-1 on tumor MDSCs is significantly higher than that of splenic MDSCs and correlates with tumor progression^26^. Tumor-infiltrating MDSCs also show differential expression of PD-L1 compared to that of spleen-derived MDSCs in mammary carcinoma, Lewis lung carcinoma, melanoma, and colon carcinoma^27^. PD-L1 expression on MDSCs during tumor infiltration and lung infiltration increases with time and is more pronounced in the late stage of tumor growth and metastasis. These data indicate that the tumor microenvironment can induce the expression of PD-1 or PD-L1 on MDSC, resulting in tumor progression.

## Effects of immune cells with high expression of PD-1 or PD-L1 in tumor immunity

### T cells

It is well-known that in many tumors, the combination of PD-L1 on tumor cells with PD-1 on activated T cells causes T cell dysfunction and thus suppresses anti-tumor immunity, which is called T cell “exhaustion”^9–12,28^. Blocking the PD-L1/PD-1 interaction can reduce the inhibitory signal and reactivate T cells, and in some cases reverse the immunodeficiency state of cancer patients. This concept has been approved for the treatment of human tumors and has been shown to provide promising antitumor responses for many patients^29^. However, many patients still develop resistance or relapse. Therefore, a better understanding of the expression and regulation of PD-L1/PD-1 in the field of tumor immunity will facilitate the development of new combinations.

A previous study reported that the production of IFN-γ by purified PD-L1^−/−^CD8^+^ T cells was significantly increased compared to wild-type (WT) CD8^+^ T cells^30^. PD-L1 induces the differentiation of Tregs by maintaining and increasing the expression of FOXP3 on Tregs^31^. These results indicate a negative regulatory function of PD-L1 in T cells. The latest results in a pancreatic ductal adenocarcinoma confirm this possibility. They verified that PD-L1^+^ T cells inhibit neighboring T cells to promote accelerated tumor growth and intratumoral immune tolerance *via* ligation of PD-1^32^. In addition, PD-L1^+^ T cells can also induce M2-like TAMs to reprogram and induce tolerogenic macrophage differentiation, which also promotes tumor growth and an adaptive immune response^32^. Collectively, this suggests that PD-L1^+^ T cells have multiple effects on innate and adaptive immune tolerances, which has great significance for the immunotherapeutic response and resistance in patients with cancer.

### B cells

Both PD-1^+^ B and PD-L1^+^ B cells have a negative immunoregulatory function on T-cell responses in diverse types of cancers. In HCC, PD-1^hi^ B cells induce T cell dysfunction through the IL10-dependent pathway, thereby creating conditions conducive to tumor progression^14^. In thyroid tumors, PD-1^+^ B cells were confirmed to be significantly increased in tumor tissue but rarely in peripheral blood^33^. PD-1^+^ B cells also inhibit the proliferation of CD4^+^ and CD8^+^ T cells in a PD-L1-dependent manner *in vitro*^33^. Notably, these PD-1^+^ B cells do not express high levels of interleukin (IL)-10. In different subtypes of breast cancers, many studies have found that tumor-induced B cells highly express PD-L1, and these cells inhibit T cell proliferation and promote the formation of regulatory T cells, which can be reversed by PD-L1 antibodies^34–37^. The latest research in glioblastomas (GBMs) showed PD-L1 overexpression on GBM-associated regulatory B cells (Bregs), which has immunosuppressive activity against activated CD8^+^ T cells. Production of the immunosuppressive cytokine transforming growth factor-β (TGF-β) and IL-10^38^ in melanomas and PD-L1^+^ B cells can suppress T cell immune responses in a PD-L1-dependent manner^15^.

Shalapour and colleagues^39^ also showed that PD-L1 is highly expressed on the surface of plasma cells with high secretion of immunoglobulin A (IgA) in prostate tumors, which induces CD8^+^ T cells to deplete or inhibit the immune response of cytotoxic T lymphocytes (CTLs). They also showed that IgA^+^ plasma cells accumulate in humans and mice with nonalcoholic steatohepatitis, and highly express PD-L1 and IL-10 and directly inhibit CTL activation as a mechanism to promote hepatocellular carcinoma^40^. Liu et al.^41^ showed that IgA^+^ B cells also accumulate in tumor tissues of colorectal cancer. Consistently, IgA^+^ B cells overexpress PD-L1 and produce a large amount of IL-10 and TGF-β and exert immunosuppressive effects on the proliferation and function of CD8^+^ T cells. Taken together, the results show that PD-L1^+^ B cells have been widely regarded as a subset of Bregs, and exert a strong immunosuppressive function on T cell responses in a PD-L1-dependent manner. However, PD-L1 may be only a functional expression, and not a specific phenotype that can characterize Bregs. Therefore, further characterization of specific transcription factors that regulate PD-L1 expression on B cells may significantly expand opportunities for tumor immunotherapy.

### NK cells

Accumulating evidence strongly suggests that PD-1 is an inhibitory regulator of NK cells in many types of tumors^42^. In ovarian cancer, PD-1^+^ NK cells exhibit low proliferative responses and impaired antitumor activities when interacting with tumor cells, which can be partially restored by blocking the PD-1/PD-L1 interaction^43^. The interaction of PD-1^+^ NK with PD-L1 on tumor cells leading to a decrease in NK cell response and a more aggressive tumor *in vivo*, has also been confirmed in many other types of cancers, including digestive cancer, melanomas, multiple myelomas, and lymphomas^17,44–46^. Once the inhibitory interaction is disrupted by PD-1 and PD-L1 blockade, the more active NK cells strongly attack tumor cells. Therefore, PD-1^−^ NK cells cannot be activated and kill tumor cells because they lack expression of PD-1. However, the cytokine cocktails tested were insufficient to induce PD-1 expression on NK cells^46^. PD-1 expression can be induced by other activation signals or a combination activation signals. Hence, identifying the mechanisms that cause NK cells to express PD-1 in tumors and the reasons for the changes of PD-1 expression in different tumors will be our main research direction in future studies.

### DCs

PD-1 is induced on DCs by various inflammatory stimuli. After lipopolysaccharide (LPS) stimulation, WT DCs increase the expression of PD-1 and start to undergo apoptosis^47^, and DCs in the spleen of PD-1 KO mice are more resistant to LPS-mediated apoptosis than those in WT mice^47^. Furthermore, PD-1-deficient DCs enhance production of antigen-specific IFN-γ and the proliferation of CD4^+^ and CD8^+^ T cells, when compared to those of WT mice^47^. In HCC, Lim et al.^48^ also showed that intratumoral transfer of PD-1-deficient DCs promotes the secretion of perforin and granzyme B by tumor-infiltrating CD8^+^ effector T cells, making the recipient mice resistant to HCC growth. These results suggest that PD-1 plays an important role in the apoptosis of activated DCs, and provides an important role for PD-1-mediated immune regulation. Moreover, PD-1^+^ DCs were found to inhibit the production of cytokines and costimulatory molecules on the cell surface and affect the antigen presentation response^19,49^.

A previous study reported that PD-L1-expressing DCs may induce the transformation of naive T cells into Foxp3^+^ Tregs and stimulate the expression of PD-1 on naive T cells, resulting in inhibition of the tumor response^50^. In a mouse model of melanoma, after apigenin treatment, DCs were incubated with cytokine-induced killer (CIK) cells, resulting in CIK cells showing robust immunity in a melanoma cell killing assay, indicating that apigenin enhances T cell immunity by limiting the expression of PD-L1 in DCs^51^.

Taken together, these results suggest that the expression of PD-1/PD-L1 on DCs hinders the survival of DCs, reduces the production of proinflammatory cytokines, inhibits innate immunity, and reduces the antitumor effect of tumor-infiltrating T cells.

### Mononuclear macrophages

PD-1 expression on TAMs inhibits phagocytosis and tumor immunity^20^. Blocking PD-1/PD-L1 *in vivo* increases the phagocytosis of macrophages, reduces tumor growth, and prolongs survival of tumor-bearing mice in a macrophage-dependent manner^20^. This is the first demonstration that PD-1 expression has a significant role in macrophages^20^. In malignant pleural mesothelioma, macrophages have a direct cytotoxic effect on mesothelioma cells, which is not related to phagocytosis^52^. However, this activity is weakened when PD-1 is overexpressed^52^. PD-1 blockade restores macrophage-dependent cytotoxicity and antitumor activity^52^.

In contrast to the role of PD-1 expression on macrophages, macrophages that overexpress PD-L1 mainly target T cells, suppressing their immune response and promoting tumor progression^21^. Monocytes exposed to tumor culture supernatants from hepatoma show significant upregulation of PD-L1 expression^21^. These activated PD-L1^+^ monocytes inhibit tumor-specific T cell immunity, and their high infiltration is associated with a low survival rate in patients with HCC^21^. Blocking PD-L1 effectively attenuates this monocyte-mediated T cell activity and restores their antitumor activity *in vivo*^21^. Another study in liver cancer showed that treatment of macrophages with exosomes from endoplasmic reticulum (ER)-stressed HCC cells increases the expression of PD-L1, and macrophages that expressed PD-L1 decrease the proportion of CD8^+^ T cells and promote T cell apoptosis^53^. In bladder cancer, PD-L1^+^ cells isolated from tumor-bearing mice also exhibit TAM morphology and express high levels of prostaglandin E2 (PGE_2_)-forming enzyme microsome PGE_2_ synthase 1, and cyclooxygenase 2 (COX2)^54^. These cells have immunosuppressive effects and are capable of eliminating CD8^+^ T cells *in vitro*^54^. Another study in melanomas also suggested that autophagosomes released by tumor cells convert macrophages into an immunosuppressive M2-like phenotype that is characterized by PD-L1 and IL-10 expression^55^. These macrophages inhibit the proliferation of T cells *in vitro* and promote tumor growth^55^. In gastric cancer, Lin et al.^56^ showed that CXCL-8 secreted by macrophages induced the PD-L1 expression on macrophages to inhibit the function of CD8^+^ T cells and promote tumor immunosuppression.

However, a recent study showed that in early stage human lung cancer, PD-L1 expressed on TAMs, in contrast to PD-L1 expressed on tumor cells, did not inhibit the interaction between tumor-specific T cells and tumor targets^57^. TAM-derived PD-L1 only plays a regulatory role when TAMs presenting related peptides interact with related effector T cells, which may limit excessive activation of T cells and protect TAMs from killing these T cells. These results suggest that the function of TAMs as primarily immunosuppressive cells might not fully apply to early stage human lung cancer, but it may explain why some PD-L1-positive patients are nonresponsive to PD-L1 therapy.

### Granulocytes

PD-1^+^ mast cells induce tolerogenic DCs with high PD-L1 and indolamine 2,3-dioxygenase expression^58^. The latter promotes the generation of Tregs with Foxp3 expression, increasing the secretion of TGF-β and IL-10 and repressing the proliferation of mitogen-stimulated naive T lymphocytes^58^. These findings indicate that mast cells can facilitate the activation of Tregs by affecting the phenotype and function of DCs, depending on the interaction of PD-1 and its ligands. There is also evidence that tumor infiltrating mast cells inhibit normal T cell immunity *via* PD-L1, and that this effect is reversed by blocking PD-L1^25^. PD-L1^+^ neutrophils also have immunosuppressive activity in T cell proliferation assays, and promote tumor progression in cutaneous melanomas and GC^24,59^.

### MDSCs

Compared with PD-1^−^ MDSC, PD-L1 and CD80 have higher expression and more proliferative activity in PD-1^+^ MDSCs^26^. PD-1 expression on MDSCs can promote tumor development and recurrence by promoting the proliferative activity of MDSCs and inducing the expression of immunosuppressive molecules^26^. Noman et al.^60^ have previously confirmed that PD-L1 expression is upregulated in MDSC under hypoxic conditions. MDSCs with high PD-L1 expression significantly increased IL-10 and IL-6 secretion of MDSC and inhibited IFN-γ production of T cells^60^. This indicates that the expression of PD-L1 increases the ability of MDSCs to alter T cell responses. In a recent study, extracellular vesicles from Ret mouse melanoma cells upregulated the expression of PD-L1 on immature myeloid cells, resulting in inhibition of T cell activation that was dependent on Toll-like receptor (TLR) expression^61^.

## Regulation of PD-1 or PD-L1 expression on immune cells in tumor immunity

**Figure 1:**
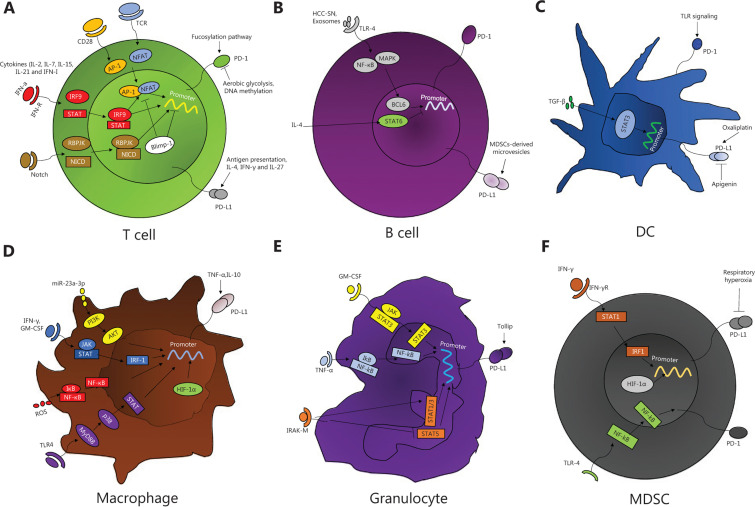
Mechanism by which PD-1/PD-L1 expression on immune cells is regulated. (A) In T cells, under TCR-mediated stimulation, NFAT drives the effector gene and PD-1 expression after association with the CD28 signaling-activated AP-1 complex. Common γ-chain family cytokines directly induce PD-1 expression and enhance the induction and maintenance of TCR-mediated PD-1 expression. The Notch signaling pathway affects PD-1 transcription by forming a complex on the PD-1 promoter. The fucosylation pathway is a positive regulator of PD-1 expression. In contrast, Blimp-1 directly binds to the PD-1 gene to inhibit NFATc1 expression or to form an inhibitory chromatin structure that inhibits PD-1 expression. DNA methylation and aerobic glycolysis are inversely related to PD-1 expression. For PD-L1, the presentation of tumor antigens and inflammatory signals induces PD-L1 expression. (B) In B cells, TLR4-mediated BCL6 upregulation is crucial for PD-1 expression. Notably, IL-4-induced STAT6 activation completely eliminates BCL6-mediated PD-1 upregulation. HCC-SN and circulating exosomes induce differentiation of PD-1^hi^ Bregs. PD-L1 expression on B cells is due to membrane-bound PD-L1 in MDSC-derived microvesicles. (C) In DCs, PD-1 is induced on DCs by various inflammatory stimuli. TGF-β increases the expression of PD-L1 by TGF-β-STAT3-PD-L1 signaling. Exposure to oxaliplatin significantly increases expression of PD-L1. However, apigenin limits PD-L1 expression in DCs. (D) In macrophages, miR-23a-3p upregulates the expression of PD-L1 by regulating the PTEN-AKT pathway. ROS activate the NF-κB signaling pathway, thereby promoting PD-L1 transcription. IFN-γ and its receptor bind to JAK, leading to STAT phosphorylation and translocation into the nucleus to regulate PD-L1 expression. TLR4-mediated MyD88-p38-STAT3 signaling stimulates PD-L1 expression. TNF-α, IL-10, and HIF-1α stimulate PD-L1 expression. (E) In granulocytes, tumor-derived GM-CSF induces PD-L1 expression *via* the JAK/STAT3 signaling pathway. IRAK-M-deficient neutrophils inhibit PD-L1 expression by reducing the activation of STAT1/3 and enhancing the activation of STAT5. Tumor-derived TNF-α induces PD-L1 expression on mast cells by activating the NF-κB signaling pathway. Tollip in neutrophils results in decreased PD-L1 expression. (F) In MDSCs, IFN-γ activates the expression of IRF1, and IRF1 binds to the unique IRF-binding consensus sequence element of CD274 promoter chromatin to directly activate PD-L1 expression. Extracellular vesicles upregulate the expression of PD-L1 in a Toll-like signaling-dependent manner. HIF-1α also stimulates the expression of PD-L1, but respiratory hyperoxia treatment inhibits the expression of PD-L1. Programmed cell death-1, PD-1; programmed cell death ligand 1, PD-L1; nuclear factor of activated T cells, NFAT; T cell receptor, TCR; activating protein-1, AP-1; interferon, IFN; interleukin 2, IL-2; interferon regulatory factor 9, IRF9; signal transducer and activator of transcription6, STAT6; Notch1 intracellular domain, NICD; recombination signal binding protein for immunoglobulin kappa J region, RBPJK; Culture supernatant from primary HCC tumor cells, HCC-SN; Toll-like receptor-4, TLR-4; B-cell lymphoma 6, BCL6; tumor necrosis factor-α, TNF-α; granulocyte-macrophage colony-stimulating factor, GM-CSF; reactive oxygen species, ROS; nuclear factor kappa B, NF-κb; myeloid differentiation primary response gene 88, MyD88; hypoxia-inducible factor-1α, HIF-1α; Janus kinase, JAK; IL-1 receptor-associated kinase M, IRAK-M.

In summary, we conclude that abnormal PD-1 or PD-L1 expression on immune cells may weaken the immunoregulatory capacity and impair T-cell responses to malignant tumors (**Table 1**). Therefore, it is important to examine how PD-1 or PD-L1 expression is regulated in activated immune cells (**Figure 1**).

### T cells

The mechanism of PD-1 expression on T cells is also relatively complicated. Under TCR-mediated stimulation, nuclear factor of activated T cells (NFAT) is dephosphorylated and transfers to the nucleus. NFAT then drives the effector gene and PD-1 expression after association with the CD28 signaling-activated activating protein-1 (AP-1) complex. Furthermore, the common γ-chain family cytokines can directly induce PD-1 expression and combined with interferon regulatory factor 9 (IRF9), can enhance the induction and maintenance of TCR-mediated PD-1 expression on T cells^68,69^. In contrast, Blimp-1 directly binds to the PD-1 gene to inhibit NFATc1 expression or to form an inhibitory chromatin structure that inhibits PD-1 expression on CD8^+^ T cells^70^.

Many studies have indicated that signaling pathways or inhibitory signals can regulate PD-1 expression on T cells^2^. The Notch signaling pathway affects PD-1 transcription in CD8^+^ T cells by forming a Notch1 intracellular domain (NICD)-recombination signal binding protein for the immunoglobulin kappa J region (RBPJK) complex on the PD-1 promoter^71^. Activated CD4^+^ T cells subjected to aerobic glycolysis show decreased PD-1 expression compared to that of T cells subjected to oxidative phosphorylation, indicating that metabolism also affects PD-1 expression^72^. DNA methylation of the gene encoding PD-1 is inversely related to the expression of PD-1in mice and humans^73^. Inhibition of Fut8, a core fucosyltransferase, reduces the expression of PD-1 on the surface of T cells and enhances T cell activation^74^.

Diskin et al.^32^ confirmed that antigen presentation drives PD-L1 expression on T cells *in vivo* and *in vitro*, which depends on Janus kinase (JAK)-signal transducer and activator of transcription (STAT) signaling. In addition, tumor-infiltrating T cells in IFN-γ-deficient and IL-27 receptor-deficient hosts express lower PD-L1. Blockade of IL-4, IFN-γ, and IL-27 can also partially reduce PD-L1 expression in Ova-restricted T cells^32^. Overall, these data indicate that the expression of PD-L1 on T cells is regulated by the presentation of tumor antigens and inflammatory signals.

### B cells

Culture supernatant from primary HCC tumor cells (HCC-SN) can induce a significant number of PD-1^+^ B cells, while culture supernatants from normal livers fail to increase PD-1 expression on B cells^14^. Blocking TLR-4/mitogen activated protein kinase (MAPK) or TLR-4/nuclear factor kappa B (NF-κB) signals successfully reduces HCC-SN-induced B-cell lymphoma 6 (BCL6) upregulation and subsequent PD-1 expression^14^. Notably, IL-4-induced STAT6 activation completely eliminates BCL6-mediated PD-1 upregulation of B cells, although it did not affect BCL6^14^. Consistent with these results, circulating exosomes from cancer patients enhance PD-1 expression in CD19^+^ B cells in ESCC^75^. Using bioinformatics technology, their analysis showed that the TLR/MAPK pathway plays a key role in regulating the transformation of PD-1^hi^ Breg^75^.

A recent study has shown that GBM-associated MDSCs deliver microvesicles transporting membrane-bound PD-L1 to B cells, thereby promoting B cell regulatory functions^38^. Functional PD-L1 *via* microvesicle transfer confers Bregs potential to inhibit CD8^+^ T cell activation and acquisition of effector phenotypes^38^. Taken together, these results are far from elucidating the mechanisms that regulate the expression of PD-1 or PD-L1 in B cells, so further clarification of its role in B cells is essential for a better understanding of tumor immunity.

### DCs

Myeloid and plasmacytoid DCs circulating in the peripheral blood do not or hardly express PD-L1. However, under TLR-induced IFN activation or stimulation, PD-L1 is upregulated^76^, which may further reduce the proliferation and cytokine secretion of T cells^77^, as well as induce the production of anergic T cells^78^. Song et al. reported that TGF-β can also upregulate PD-L1 expression in DCs through STAT3, thereby suppressing T cell-mediated immunity^62^ and maintaining Treg cell populations^79^. Another study showed that exposure to oxaliplatin increased PD-L1 on TLR9-activated human DCs, resulting in reduced T cell proliferation^80^. Therefore, current DC-based immunotherapies can be used by interfering with the PD-1/PD-L1 signaling pathway according to the expression and regulation pattern of PD-1/PD-L1 on DCs^79^.

### Mononuclear macrophages

In gliomas, upregulation of PD-L1 expression in circulating monocytes and tumor infiltrating macrophages yield an immunosuppressive phenotype by regulating autocrine/paracrine IL-10 signaling^63^. In melanomas, proinflammatory mediators released by tumors promote tumor necrosis factor (TNF)-α production by monocytes in a TLR-2-dependent manner and increase the PD-L1 expression on the surface of monocytes^81^. Wen et al.^55^ reported that the expression of PD-L1 induced by tumor cell-released autophagosomes (TRAPs) is completely myeloid differentiation primary response gene 88 (MyD88)-dependent, and the upregulation of PD-L1 is significantly weakened because of the lack of TLR4 instead of TLR2. Further results suggested that the polarization of macrophages is caused by protein components on TRAPs through activation of the p38-STAT3 pathway.

In TNBC, treatment of macrophages with reactive oxygen species (ROS) inducers (such as paclitaxel) results in a corresponding increase in PD-L1 expression^64^. This is because these drugs cause an increase in ROS, leading to activation of the NF-κB signaling pathway, thereby promoting PD-L1 transcription and release of immunosuppressive chemokines^64^. Thus, combination therapy with paclitaxel and PD-L1 antibodies reduces tumor burden and increases the number of CTLs^64^.

Another study^65^ in TNBC showed that tumor cells can stimulate PD-L1 expression on macrophages by using the JAK2/STAT3 signaling pathway. G-CSF treatment can increase JAK2 phosphorylation and upregulate PD-L1 levels in CD169^+^ macrophages^65^. In bladder cancer, tumor cells affect the metabolism of PGE_2_ in bone marrow-derived myeloid cells, driving their differentiation into PD-L1^+^ macrophages^54^. Inhibition of PGE_2_ production *in vivo* significantly reduces PD-L1 expression on TAM^54^, and targeting the metabolism of PGE_2_ can help reduce PD-L1-mediated immune suppression^54^. In liver cancer, due to the high level of miR-23a-3p in HCC cell-derived exosomes induced by ER stress, miR-23a-3p upregulates the PD-L1 expression in macrophages by regulating the phosphatase and tensin homolog (PTEN)-AKT pathway^53^.

### Granulocytes

In GC, tumor-derived granulocyte-macrophage colony-stimulating factor (GM-CSF) activates neutrophils and induces PD-L1 expression on neutrophils *via* the JAK/STAT3 signaling pathway^24^. Nevertheless, tumor-derived TNF-α induces PD-L1 expression on mast cell by activating the NF-κB signaling pathway^25^.

In a chemically induced colorectal cancer model, selective removal of Tollip (a key innate immune cell modulator) in neutrophils results in enhanced CD80 expression and decreased PD-L1 expression^66^. Adoptive transfer of Tollip^−^/^−^ neutrophils is sufficient to enhance tumor immune surveillance and reduce the burden of colorectal cancer *in vivo*^66^. Another study showed that IL-1 receptor-associated kinase M (IRAK-M) mediates the immunosuppressive effects of neutrophils on T cell proliferation and activation by reducing CD80/CD40 and enhancing PD-L1^67^. Adoptive transfer of IRAK-M^−^/^−^ neutrophils can inhibit colitis-associated tumor progression^67^.

### MDSCs

LPS induces PD-1 expression on MDSCs through the NF-κB signaling pathway, as do macrophages^26,82^. Hypoxia induces a significant, rapid, and selective upregulation of PD-L1 on splenic MDSCs of tumor-bearing mice, which depends on hypoxia-inducible factor (HIF)-1α^60^. This effect is not limited to MDSCs, but also occurs on macrophages, dendritic cells, and tumor cells^60^. Respiratory hyperoxia treatment improves hypoxia levels in the lung tumor microenvironment, and reduces the proportion of MDSCs and PD-L1 expression in primary tumors and metastatic lungs, thereby enhancing antitumor T cell immunity^27^. It was also confirmed in another study that MDSCs accumulate abundantly in the peripheral blood of patients with microsatellite instability and microsatellite stable colon cancer, and a large proportion of these MDSCs are PD-L1^+83^. The mechanism involves IFN-γ induction of the expression of IRF1, IRF5, IRF7, and IRF8 in MDSCs, but only IRF1 binds to the unique IRF-binding consensus sequence element of CD274 promoter chromatin to directly activate PD-L1 expression in MDSCs^83^. Neutralization of IFN-γ reduces PD-L1 expression on MDSCs^83^.

## Potential therapeutic strategies of anti-PD-L1/PD-1 therapy

Although anti-PD-L1/PD-1 therapy has shown durable clinical responses in many types of tumor patients, their drug resistance is still widespread^57^, involving the emergence of new immune escape pathways and gene mutations in tumor cells^84^. It is therefore necessary to elucidate the role of PD-1 or PD-L1 in the tumor microenvironment for better curative effects of anti-PD-L1/PD-1 therapy, as well as to identify new methods of tumor immunotherapy^85^.

**Table 1** summarizes the effects of immune cells that express PD-1 or PD-L1 in solid tumors. Although T cells are the main targets of inhibition, the PD pathway may also impair the anti-tumor function of innate immune cells through a PD-L1/PD-1 counter-receptor interaction, including DCs, macrophages, and NK cells. Compared with adaptive immune cells, PD-1 mainly works in innate immune cells in a unique way. However, PD-L1 expression on almost all types of immune cells plays an immunosuppressive and pro-tumor role in many types of tumors. Furthermore, PD-L1/PD-1-mediated inhibition mechanisms seem to be complicated^2^, including the induction of inhibitory cytokine factors, proliferation, apoptosis, anergy, and Tregs production. Thus, the mechanisms by which these effects are exerted are being studied in depth. We should therefore further explore the mechanisms that exert these effects in order to improve the efficacy of anti-PD antibodies.

Secondly, the above data indicate that there are also many mechanisms regulating PD-1/PD-L1 expression on immune cells (**Figure 1**). The expression of PD-1 or PD-L1 can be regulated by a large number of inflammatory mediators, basic transcription factors, effector components of signaling pathways, and oncogenic signals altered by upstream receptors. Anti-PD-L1/PD-1 therapy has therefore become the center of solid tumor immunotherapy and the cornerstone of combined immunotherapy^4,86^. We envisage that the targets of these mechanisms can be combined with anti-PD antibodies to produce a synergistic effect to improve the efficacy of anti-PD antibodies and form new treatment combinations to enhance anti-tumor efficacies. For example, activated T cells or innate immune cells can release type I and type II IFN to stimulate PD-L1 expression. In a mouse colorectal cancer model, the combined use of IFN-α and anti-PD-1 therapy can increase tumor infiltration of CD4^+^ and CD8^+^ T cells and inhibit tumor growth, which is better than IFN-α alone^62^. Also, HIF-1α regulates PD-L1 expression by directly binding to HIF-1α site in the PD-L1 proximal promoter. The combined treatment of HIF-1α inhibitors and PD-L1 blockade against tumor hypoxia may be beneficial for strengthening the immune system of cancer patients^60^. These results could provide a theoretical basis for the combination of immune checkpoint inhibitors and other therapies.

## Conclusions

Current evidence suggests that immune cells expressing PD-1 or PD-L1 play an important role in tumor immunity. It is therefore important to extensively study the expression of PD-L1/PD-1 and related regulatory mechanisms, especially on immune cells, which will help to design new clinical treatments. In future studies, many aspects remain to be clarified, including the following: (1) whether cytokines stimulate the upregulation of PD-1 or PD-L1 in an indirect manner, for example, upregulating the production of IFN; (2) if the same signal pathway components have different effects in different cancer species; (3) whether a large number of *in vitro* experiments are reproducible *in vivo*; and, (4) whether the subtypes of PD-1^+^ or PD-L1^+^ immune cells have their own role, and which effect is dominant.

## Figures and Tables

**Table 1:** Characteristics of immune cells expressing PD-1/PD-L1 in tumors immunity

Cell types	Phenotypes	Target cells	Effects	Cancer types	References
T cells	PD-1^+^CD8^+^ T cell	Tumor cells	Induce T cell exhaustion	Liver, pancreatic, HNC, NSCLC, early breast cancer	^9–12^
	PD-L1^+^ T cell	Neighboring effector T cells, macrophages	Promote STAT6-dependent M2-like macrophage differentiation and suppress neighboring effector T cells	Pancreatic ductal adenocarcinoma	^32^
B cells	PD-1^hi^CD5^hi^CD24^-/+^CD27^hi/+^CD38^dim^ B cell	T cells	T-cell dysfunction and foster disease progression	Hepatoma	^14^
	PD-1^+^ B cell	CD4^+^ and CD8^+^ T cells	Inhibit CD4^+^ and CD8^+^ T cell proliferation	Thyroid tumors	^33^
	PD-L1^hi^ B cell, PD-1^-^PD-L1^+^CD19^+^, PD-L1^+^CD24^+^CD38^+^CD19^+^ B cell	T cells	Inhibit T cell proliferation and promote the formation of Tregs	Breast cancer	^34–37^
	PD-L1^+^CD155^+^IL-10^+^TGF-β^+^ B cell	CD8^+^ T cells	Inhibit CD8^+^ T cell proliferation, IFN-γ production, and expression of the granzyme B	GBM	^38^
	PD-L1^+^IgA^+^IL-10-producing Plasmocyte	CD8^+^ T cells, CTLs	Induce CD8^+^ T cell depletion and inhibit the immune response of CTLs	Prostate tumors	^39^
	PD-L1^+^IgA^+^IL-10^+^ Plasma cell	CD8^+^ T cells	Inhibit activation of CTLs	Hepatocellular carcinoma	^33^
	PD-L1^+^IgA^+^CD19^+^ B cell	CD8^+^ T cells	Inhibit the proliferation and activation of CD8^+^ T cells	Colorectal cancer	^40^
NK cells	PD-1^+^NKG2A^-^KIR^+^CD57^+^ NK cell	Tumor cells	Low proliferative response of NK cells and impaire antitumor activity	Ovarian cancer, colorectal cancer, gastric cancer, ESCC, HCC, biliary cancer, melanoma	^17,44–46^
DCs	PD-1^+^CD11c^+^ DC	CD8^+^ T cells	Inhibit the secretion of perforin and granzyme B by CD8^+^ T cells	HCC	^48^
	PD-1^+^PD-L1^+^CD11c^+^CD8α^-^Gr-1^ lo / int^ DC	T cells, DC cells	Inhibit T cell proliferation, regulate cytokine production by DCs and maintain their immature phenotype	Ovarian cancer	^19^
	PD-L1^+^ DC	CIK cells	Reduce the ability of DC-CIK-mediated melanoma cell killing assays	Melanoma	^51^
	PD-L1^+^ DC	T cells	Induce the apoptosis of T cells and increase the percentage of Tregs	HCC	^62^
Mononuclear macrophages	PD-1^+^CD11c^+^MHC-II^+^ CD4^+^CD68^+^ TAM	Tumor cells	Decreased phagocytosis	Colorectal cancer	^20^
	PD-1^+^ Macrophage	Tumor cells	Weaken the cytotoxic effect on mesothelioma cells	Malignant pleural mesothelioma	^52^
	PD-L1^+^HLA-DR^high^CD68^+^ Macrophage	T cells	Induce T cell anergy and reduce IFN-γ production by T cells	HCC	^21^
	PD-L1^+^ CD68^+^ Macrophage	T cells	Decrease the proportion of CD8^+^ T cells and promote T cell apoptosis	HCC	^53^
	PD-L1^+^F4/80^+^ Macrophage	CD8^+^ T cells	Reduce the number of activated T lymphocytes	Bladder cancer	^54^
	PD-L1^+^CD11b^+^F4/80^+^ Macrophage	CD4^+^ and CD8^+^ T cells	Inhibit CD4^+^ and CD8^+^ T cell proliferation	Melanoma	^55^
	PD-L1^+^CD45^+^CD11b^+^ Macrophage	T cells	Induce T cell apoptosis	Glioma	^63^
	PD-L1^+^CD11b^+^F4/80^+^ TAM CD169^+^ Macrophage	T cell	Inhibit T cell-mediated antitumor response	TNBC	^64,65^
	PD-L1^+^CXCL8^+^CD68^+^CD45^+^ Macrophage	CD8^+^ T cells	Inhibit CD8^+^ T cells function	Gastric cancer	^56^
	PD-L1^+^HLA-Dr^hi^CD14^+^ TAM	T cells	Protect TAMs from being killed by cognate effector T cells	Early-stage lung tumor	^57^
Granulocytes	PD-L1^+^CD45^+^CD117^+^Fc?RI^+^ Mast cell	T cells	Inhibit normal T cell immunity	Gastric cancer	^25^
	PD-L1^+^CXCL5^+^CXCR4^+^ CCR5^+^Adam1^+^Nos2^+^Neutrophil	T cells	Immunosuppressive effects in T cell proliferation	Cutaneous melanoma	^59^
	PD-L1^+^ CD54^+^ Neutrophil	T cells	Suppresse normal T-cell immunity	Gastric cancer	^24^
	PD-L1^+^Tollip^+^ Neutrophil	T cells	Reduce T cell activation, suppress tumor immune surveillance and increase the tumor burden	Colorectal cancer	^66^
	PD-L1^hi^CD11b^hi^CD80^low^IRAK-M^+^ Neutrophil	T cells	Inhibit T cell activation	Colitis-associated tumor	^67^
MDSCs	PD-L1^+^Gr-1^+^CD11b^+^ MDSC	MDSCs, T cells	Increase MDSC-mediated T cell suppression, increased IL-10 and IL-6 secretion of MDSC and inhibited IFN-γ production of T cells	Mammary carcinoma, LLC, melanoma, colon carcinoma	^27^
	PD-L1^+^Gr-1^+^CD11b^+^ MDSC	T cells	Inhibit T cell activation	Melanoma	^61^
